# Chronic hyperkalemia in non-dialysis CKD: controversial issues in nephrology practice

**DOI:** 10.1007/s40620-018-0502-6

**Published:** 2018-06-07

**Authors:** Luca De Nicola, Luca Di Lullo, Ernesto Paoletti, Adamasco Cupisti, Stefano Bianchi

**Affiliations:** 1 Division of Nephrology, University of Campania, Piazza L. Miraglia, 1, 80138 Naples, Italy; 2 Nephrology and Dialysis Unit, Parodi-Delfino Hospital, Colleferro, Rome, Italy; 3 Nephrology, Dialysis and Transplantation, University of Genoa and Policlinico San Martino, Genoa, Italy; 4 Department of Clinical and Experimental Medicine, University of Pisa, Pisa, Italy; 5 Nephrology Unit, Azienda USL Toscana Nord Ovest, Leghorn, Italy

**Keywords:** Hyperkalemia, Chronic kidney disease, Anti-RAAS, Potassium binder

## Abstract

Chronic hyperkalemia is a major complication of chronic kidney disease (CKD) that occurs frequently, heralds poor prognosis, and necessitates careful management by the nephrologist. Current strategies aimed at prevention and treatment of hyperkalemia are still suboptimal, as evidenced by the relatively high prevalence of hyperkalemia in patients under stable nephrology care, and even in the ideal setting of randomized trials where best treatment and monitoring are mandatory. The aim of this review was to identify and discuss a range of unresolved issues related to the management of chronic hyperkalemia in non-dialysis CKD. The following topics of clinical interest were addressed: diagnosis, relationship with main comorbidities of CKD, therapy with inhibitors of the renin–angiotensin–aldosterone system, efficacy of current dietary and pharmacological treatment, and the potential role of the new generation of potassium binders. Opinion-based answers are provided for each of these controversial issues.

## Introduction

In 2007, the National Kidney Foundation launched the message that non-dialysis chronic kidney disease (CKD) is “common, harmful, and treatable” [1]. The purpose of this message was to raise awareness of CKD in order to improve diagnosis and treatment of this high-risk condition. Since then, the population of CKD patients referred to renal clinics has grown considerably, in recognition of the value of conservative treatment of CKD, even in advanced stages of the disease [2]. To paraphrase the afore-mentioned message, it is possible to state to a similar extent that hyperkalemia is a major complication of CKD, because it is frequent, associated with poor prognosis and potentially controlled by adequate therapy.

The prevalence of hyperkalemia reported in the most recent observational studies ranges from 1 to 50%, and is mainly dependent on the threshold level of serum potassium (sK) used for diagnosis (Table 1) [3–12]. Moreover, prevalence strictly correlates with residual renal function; indeed, the kidney is the main regulator of potassium homeostasis, and impaired renal function is the essential prerequisite for the development and maintenance of chronic hyperkalemia [13]. Additional key modifiers of the hyperkalemia rate are comorbidities frequently encountered in the CKD population, namely diabetes and heart failure (HF), and use of the first-line agents for treatment of CKD, namely renin–angiotensin–aldosterone system antagonists (anti-RAAS), particularly if used in combination [13].

**Table 1:** Main observational studies published in the last 5 years on prevalence of hyperkalemia by eGFR

Study [Ref.]	Setting	Sample size	eGFR ml/min/1.73 m^2^	Hyperkalemia prevalence, % *(definition)*	Survival by sK level
Hayes [3]	Renal clinic	1227	37.0	7.7 (*sK* > 5.3)	↓ for sK > 5.5
Drawz [4]	Veterans hospital	13,874	46.5	2.5 (*sK* ≥ 5.5)	Not available
Sarafidis [5]	Low-GFR clinic	238	14.5	54.2 (*sK* > 5.0) 31.5 (*sK* ≥ 5.5) 8.4 (*sK* ≥ 6.0)	Not available
Nakhoul [6]	CKD registry at Cleveland clinic	36,359	47.0	11.0 (*sK* > 5.0) 3.3 (*sK* > 5.5)	↓ for sK > 5.0
Luo [7]	Health care system	55,266	< 60	14.9 (*sK* 5.0–5.4) 3.9 (*sK* 5.5–5.9) 1.1 (*sK* ≥ 6.0)	↓ for sK > 5.0
Chang [8]	Health care system	155,695	< 60	5.3, 19.0, 9.4 for < 2, 2 to < 4, ≥ 4 sK test/year (*sK* > 5.0)	Not available
Hughes [9]	General population	9651	80.0	2.8 *(sK* ≥ 5.0)	↓ for sK ≥ 5.0
Betts [10]	Medicare 2014	120,933	< 60	9.4 in CKD 3 19.5 in CKD 4 18.5 in CKD 5 52.8 in dialysis (*sK* > 5.0)	Not available
Núñez [11]	Heart failure	2164	62.0	5.6 (*sK* > 5.0)	↓ for sK > 5.0
Collins [12]	US integrated health delivery networks	911,698	with and w/o CKD (eGFR < 60)	8.3 in DM 9.1 in HF 11.5 in CKD 13.1 in combined (*sK* ≥ 5.0)	↓ for sK ≥ 5.0

*CKD* chronic kidney disease, *eGFR* estimated glomerular filtration rate, *sK* serum potassium

The relatively large prevalence of hyperkalemia in patients under stable nephrology care, and even in the ideal setting of randomized trials where best treatment and monitoring are mandatory, indicates that current strategies aimed at prevention and treatment of hyperkalemia are suboptimal.

The therapeutic issue becomes even more critical when considering the prognostic value of high sK levels. Indeed, the excess risk of mortality already becomes evident for even minor degrees of hyperkalemia (Table 1), suggesting that sK ≥ 5.0 mEq/l should receive the attention of nephrologists, because it likely poses future risk of further increases in sK to levels triggering fatal arrhythmias. Furthermore, it is possible that hyperkalemia may play an indirect role in the progression of CKD to dialysis by dictating the withdrawal of nephroprotective anti-RAAS agents [8]. Finally, hyperkalemia represents a main driver for dialysis initiation. In this regard, a recent survey among European nephrologists revealed that the clinical picture plays the decisive role, with hyperkalemia refractory to medical therapy eliciting preference for an immediate start of dialysis by 100% of nephrologists participating in the survey [14]. These results are coherent with contemporary guidelines that, while not identifying a specific estimated glomerular filtration rate (eGFR) level to start dialysis in stage 5, recommend it when complications become uncontrolled by conservative therapy [15].

Therefore, on the understanding that hyperkalemia is a main refractory complication of CKD, and that the presence of hyperkalemia per se markedly increases the economic expenditure for CKD due to more frequent and costly hospitalizations [16], we here address some major controversial issues in the clinical management of hyperkalemia and provide opinion-based answers.

### Number of blood samples needed to diagnose hyperkalemia in CKD

No consensus exists on how many tests showing an increase in sK are needed to identify a clinically meaningful hyperkalemia. Current Kidney Disease Improving Global Outcomes (KDIGO) guidelines for the evaluation and management of CKD only suggest sK measurement within 1 week of starting anti-RAAS, or uptitrating dose, in subjects with reduced renal function [15]. Similarly, NICE guidelines [17] recommend sK testing in CKD patients administered anti-RAAS before starting therapy and between 1 and 2 weeks thereafter and after each dose increase. Independently from anti-RAAS prescription, most observational studies have evaluated the prevalence of chronic hyperkalemia on the basis of a single sampling [6, 18, 19], whereas recent randomized controlled trials (RCTs) performed sK laboratory tests at least twice in order to identify subjects that could be enrolled for active treatment of hyperkalemia [20–22].

A recent study including CKD patients showed that detection of hyperkalemia increased as the number of sK tests increased [8]. In particular, in patients with eGFR ranging from 30 to 44 ml/min/1.73 m^2^ and more than 4 laboratory tests/year available over a 3-year observation period, sK > 5 mEq/l was found in 54% while 22% had sK > 5.5 mEq/l. Furthermore, the prevalence of hyperkalemia significantly increased with lower eGFR and the use of anti-RAAS. Of note, in the Renal Research Institute CKD Study cohort [23], an average of six sK values was obtained during a 2.6-year follow-up period in CKD patients in order to better identify subjects with hyperkalemia (sK ≥ 5.5 mEq/l).

Therefore, although single sK testing can identify patients at risk for chronic hyperkalemia, especially those with more advanced CKD and those administered anti-RAAS, testing at least two times per year seems to be a wise approach to properly identify chronic hyperkalemia. Assessment of sK in CKD patients becomes essential before starting anti-RAAS therapy, 1–2 weeks thereafter, and at each uptitration of dose.

### Electrocardiogram for the stratification of hyperkalemia severity

**Fig. 1:**
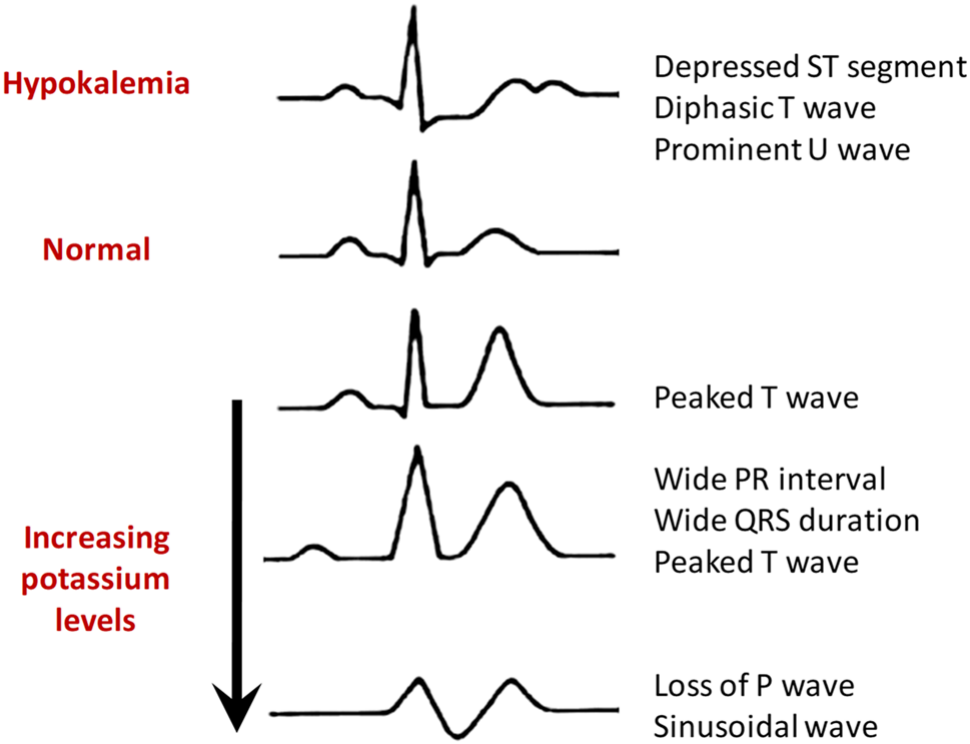
Changes in electrocardiogram following progressive increases in potassium levels

Chronic hyperkalemia is easily diagnosed by common laboratory tests, paying attention to exclude pseudohyperkalemia (Table 2) [24]. Bradycardia is an early sign of acute severe hyperkalemia that requires increased awareness, especially in the dialysis setting [25]. In chronic hyperkalemia, where bradycardia is uncommon because of adaptive mechanisms, electrocardiogram (ECG) monitoring allows to gain clinical insight into the severity of abnormalities related to sK-dependent changes in cardiac rhythm (Fig. 1).

**Table 2:** Determinants of pseudohyperkalemia

Cause	Explanation
Mechanical factors	Tourniquet applied for prolonged periods, fist clenching, traumatic venipuncture or probing, vigorous mixing or excessive centrifugal force of specimens
Chemical factors	Ethanol containing antiseptics
Temperature	Specimens exposed to low (2–8 °C) or above room temperature can result in potassium leakage
Time	Delayed processing can result in potassium leakage
Patient factors/diseases	Fear of venipuncture/crying and associated with hyperventilation (even for a few minutes) is associated with acute respiratory alkalosis and significant hyperkalemia. Thrombocytosis and chronic lymphocytic leukemia can result in increased potassium levels
Miscellaneous	False plasma reference ranges or mislabelling, contaminants such as potassium containing intravenous fluids

The progressive changes of hyperkalemia are classically listed as follows:

- ST-segment depression
- widening of the PR interval
- widening of the QRS interval
- loss of the P-wave
- a sine-wave pattern—an ominous development and a harbinger of impending ventricular
- fibrillation and asystole [26].

However, sK levels often poorly correlate with cardiac manifestations. In a retrospective review, only 16 of 90 cases met criteria for ECG changes reflecting hyperkalemia (defined as new peaked and symmetric T waves that resolved on follow-up) [27]. In 13 of these cases, the ECG was interpreted as showing no T-wave changes even when read by a cardiologist. In addition, criteria for hyperkalemia were only noted in 1 of 14 patients who manifested arrhythmias or cardiac arrest attributed to increased sK concentration. Therefore, ECG can only be used as a support to stratify risk in patients with severe hyperkalemia, as previously suggested [28]. Indeed, although ECG is considered a main diagnostic tool for hyperkalemia diagnosis, this is true in only 40–50% of chronic hyperkalemic patients.

### Major risk factors for newly diagnosed hyperkalemia in CKD

Among CKD-related hyperkalemia risk factors, HF is widely represented as it is frequently associated with low GFR [29]. Furthermore, patients with HF and CKD are extensively treated with anti-RAAS, and in particular with mineralocorticoid receptor antagonists (MRAs), that are major contributors to hyperkalemia onset [30]. Notably, in HF, hyperkalemia can also be related to reduced distal sodium delivery and GFR worsening, comorbidities other than CKD (diabetes mellitus, Addison’s disease, hypoaldosteronism), as well as nonsteroidal anti-inflammatory drugs, and angiotensin receptor neprilisin inhibitors (ARNIs) [31].

In the PARADIGM-HF trial, approximately 15% of patients developed hyperkalemia in both treatment arms (ARNI and enalapril) [32]. Similar findings were observed in the RALES trial where a higher risk of mortality was observed with sK > 5.5 mmol/l [33]. Finally, in the ADHERE study, more than 60% of patients presented with kidney disease, and up to 20% developed hyperkalemia [7, 34].

The incidence of hyperkalemia is also higher in diabetic patients [31]. Redistribution of potassium from the intracellular to the extracellular compartment can induce hyperkalemia with no net total body K^+^ increase, as occurs in acidosis (for each 0.1 fall in pH, sK increases by approximately 0.4 mmol/l), insulin deficiency, hypertonicity, cell lysis (rhabdomyolysis), and drugs (e.g. beta blockers). The most common causal factor of chronic hyperkalemia in diabetics is the reduced tubular secretion of K^+^ due to the hyporeninemic hypoaldosteronism syndrome [35]. Interestingly, the new antidiabetic drugs (e.g. sodium-glucose co-transporter-2 inhibitors) may actually protect patients from hyperkalemia due to the osmotic diuresis [35].

Therefore, CKD patients with HF and/or diabetes are at high risk of developing chronic hyperkalemia. In these patients, chronic hyperkalemia is often related to concomitant treatments (i.e. anti-RAAS, nonsteroidal anti-inflammatory drugs and ARNI) over and above the frequent concurrent GFR impairment.

### Nutritional management in CKD patients at risk of hyperkalemia

In CKD patients, dietary interventions are prescribed in order to delay the start of dialysis [36], both in proteinuric and in non-proteinuric kidney disease [37, 38]. Diets are generally based on control/reduction of the intake of sodium, phosphate and proteins while providing an adequate supply of essential amino acids. A major point is that energy intake must cover the energy requirement (30–35 Kcal/kg/day) to allow metabolic adaptation in the course of protein-restricted regimens, thus preventing negative nitrogen balance and protein-energy wasting. Diets rich in plant-based foods, as vegan or vegetarian regimes [39], provide additional favorable effects related to high amounts of alkali and fibers [40]. Lower net acid load and favorable effects on intestinal motility and microbiota are expected, but the resulting high potassium load is of concern.

Therefore, when protein restriction is needed in a CKD patient, an animal-based low-protein diet (LPD) could be favored over vegetarian-based LPDs [39].

However, the prevention or correction of metabolic acidosis as well as constipation may well counteract the hyperkalemia-inducing effects of high potassium intake. This may explain why an increase in sK or overt hyperkalemia in CKD patients is a rare finding in patients on vegetarian diets, with or without low protein content [41–44].

In patients with non-dialysis dependent CKD stages 1–5, the National Kidney Foundation suggests an unrestricted potassium intake (90–120 mmol/day) as for the recommended daily allowance in the general population unless hyperkalemia occurs [45]. Potassium intake should be limited to 2.7–3.1 g/day in hemodialysis patients and to 3–4 g/day in peritoneal dialysis patients; in either case, adjustment based on sK level is necessary. A recent comprehensive review has suggested an intake of 4.7 g/day in the early stages of CKD without risk of hyperkalemia, while dietary potassium restriction down to 2–3 g/day (approximately 51–77 mmol/day) is recommended in CKD patients with sK > 5.3 mEq/l [46].

Correct dietary counseling and intervention are therefore crucial in CKD patients in order to obtain clinical and metabolic beneficial effects together with good nutritional status while limiting dietary potassium load in the cases of chronic or recurrent hyperkalemia.

Careful recall of potassium intake may focus the dietary intervention and prevent excessive potassium load, leading to a reduction in sK. Unfortunately, education for a dietary approach to limiting potassium-rich foods is uncommon during clinician visits. Studies on the effectiveness of potassium dietary management in patients with advanced CKD are needed because up to now, the efficacy of dietary counseling in the prevention or treatment of hyperkalemia remains to be demonstrated [47].

In summary, safe nutritional intakes in CKD patients at risk of hyperkalemia include: protein intake of ≤ 0.8 g/kg/day; salt intake of 5–6 g/day; phosphate intake of ≤ 700 mg/day; energy intake of 30–35 Kcal/kg/day; potassium intake of 2–3 g/day and alkali and fiber intake of 25–30 g/day.

### Control of potassium load while maintaining the benefit of a high-potassium diet

A dietary potassium intake that surpasses output, i.e. a positive external potassium balance, plays a central role in the pathogenesis of chronic hyperkalemia [48]. Net intestinal absorption of dietary potassium is approximately 90%. It is absorbed mostly in the duodenum and jejunum, and fecal excretion is constant at about 10 mmol/day, with a maximum level of 15–20 mmol/day.

Potassium concentration in the feces is very high (83–95 mmol/l) and the capacity of the colon/rectum to secrete potassium seems to be increased at very low levels of residual kidney function [49]. Slow fecal transit time along the intestinal tract favors potassium absorption, whereas faster intestinal transit time reduces potassium absorption [50]. This has led to the hypothesis that constipation, more than potassium dietary load, acts as the main determinant of hyperkalemia in end-stage renal disease (ESRD) patients [51].

In the early stages of CKD, patients maintain an ample capacity to eliminate high potassium load from the diet, so hyperkalemia does not usually occur and the external potassium balance is generally neutral, unless therapies changing the internal balance or impairing renal excretion capacity are administered. This is a crucial point, because high potassium diets have been associated with positive cardiovascular (CV) and renal outcomes in CKD patients [52]. Hence, proper dietary counseling is mandatory in CKD patients with chronic hyperkalemia as in those with sporadic hyperkalemic episodes [48].

Special attention must also be paid to avoid excess potassium load, together with the maintenance of a high fiber intake and a low net fixed-acid production. This is important since constipation and metabolic acidosis are major risk factors for chronic hyperkalemia [51].

In order to achieve a lower potassium intake without a decrease in fiber and alkali intake, some home-based low-cost pragmatic suggestions may be provided. The first step is the availability and awareness of information on the type of foods which contain excess potassium for serving or for unit of weight, which should be avoided. Another tool for food selection may be based on the potassium content normalized for unit of fiber, namely the reporting of the potassium content of vegetables and fruits also as “mg per 1 g fiber” [48]. An additional suggestion to limit potassium intake is the use of cooking procedures (e.g. soaking or boiling) that allow food demineralization: boiling can remove up to 60–80% of potassium content from several raw foods [53, 54].

Attention should be paid to hidden sources of potassium, such as certain food additives [55] and, in particular, salt substitutes. The latter represents a significant potassium load on top of the potassium coming from food, which is of concern in patients with reduced capacity of potassium excretion. In low-sodium salts, sodium-chloride content must not exceed 35% (corresponding to a sodium content not > 13.6%) and cannot be less than 20% (corresponding to a sodium content not < 7.8%) with a potassium:sodium ratio of 1.5:1 at least. This means that 5–6 g of a current salt substitute can supply up to 1500–1800 mg (38–46 mmol) of extra-potassium [48].

Although no human study is available to date, dietary counseling can be effective for the prevention and treatment of hyperkalemia. From a pathophysiology basis, the limitation of potassium intake counteracts the tendency to positive external potassium balance in CKD [47, 56]. It is conceivable that the effective reduction of dietary potassium intake is an important tool in the treatment of chronic hyperkalemia; however, it is not common practice because it is time-consuming and difficult to perform in a real-life clinical setting. Poor dietary education given to health care providers is a frequent additional reason [47].

Pragmatic interventions to lower potassium load without inducing a decrease in alkali or fiber intake, therefore, are: (a) identify foods that contain excess potassium load, (b) prefer foods that supply a low amount of potassium both as absolute value and as normalized per unit of fiber content, (c) educate patients on the use of cooking procedures (especially boiling) in order to remove potassium, and (d) avoid hidden sources of potassium (e.g. food additives and low-sodium salt substitutes).

### Rationale for intensive (and potentially hyperkalemic) anti-RAAS therapy

Current nephrology guidelines recommend anti-RAAS in patients with urine albumin excretion > 300 mg/24 h or proteinuria > 500 mg/24 h [15]. This “1B” recommendation is based on the proven nephroprotective efficacy of these agents in proteinuric patients. A subsequent meta-regression analysis has supported this rationale by revealing that the risk of ESRD decreases by 24% for each 30% reduction in albuminuria, with clinical meaningful efficacy observed for anti-RAAS [57]. The positive effect of these drugs in delaying ESRD has also been verified by a network meta-analysis of randomized clinical trials [58], and even confirmed in advanced disease [59]. It is worth noting that anti-RAAS also affords a survival benefit; analyses in a large cohort of older patients, most with CKD stage 3 and albuminuria, have in fact shown that anti-RAAS therapy yields a 19% lower risk of mortality [60]. This finding extends to overt CKD; the post-hoc analysis of the LIFE trial in hypertensive individuals with mild CKD showed that the level of CV protection was proportional to the degree of albuminuria reduction [61]. Interestingly, anti-RAAS agents can also improve CV prognosis in non-albuminuric patients [62, 63]. Conversely, in the growing population of patients with proteinuria less than 0.5 g/24 h [64], the nephroprotective effects of anti-RAAS are less evident or even absent [65, 66].

Therefore, anti-RAAS drugs are the primary therapeutic tools used to improve renal prognosis in proteinuric patients, as indicated by current guidelines [15]. Experimental studies have indeed demonstrated that albumin tubular overload sustains a sequence of inflammatory events leading to tissue scarring and eGFR loss, while anti-RAAS delays progression of kidney damage and dysfunction by lowering glomerular pressure and restoring glomerular sieving function. With regard to the association with cardiovascular risk, it is possible that albuminuria may simply act as a marker of endothelial dysfunction [67–69].

Guidelines identify a proteinuria level of 0.5 g/24 h as a major goal of antiproteinuric therapy [15]. Nevertheless, almost half of patients under stable nephrology care including anti-RAAS therapy do not reach this target in renal clinics despite evidence that even residual proteinuria portends a significant excess risk of ESRD [70, 71]. Therefore, while nephrologists should pursue the goal of minimizing proteinuria, this target is de facto hardly achievable.

Two major reasons account for this therapeutic failure. First, side effects of anti-RAAS restrain their use. Specifically, a clinically significant acute kidney injury (AKI) in the course of anti-RAAS monotherapy, which is dependent on GFR worsening due to underlying (and often undiagnosed) ischemic nephropathy, occurs in less than 2% of CKD patients, particularly those under diuretics [72]; however, incidence rates markedly increase in the presence of dual blockade of RAAS [73]. Hyperkalemia is more common than AKI with an incidence ranging from 5 to 40%, and, moreover, it acts as a main driver for anti-RAAS withdrawal [8, 13, 30].

Second, the high complexity of the RAAS with multiple-level escape mechanisms prevents adequate suppression by anti-RAAS monotherapy that allows only a 30% reduction on average. Multiple-level blockade of RAAS has been in fact tested to overcome escape pathways. This approach, though more effective in reducing albuminuria, and probably the ESRD risk as well, is associated with a slightly greater risk of AKI and can even double the risk of hyperkalemia versus single-drug therapy [74–77]. An alternative option to dual blockade in diabetic CKD may be the association of a single anti-RAAS agent with an inhibitor of renal sodium-glucose cotransporter-2 (SGLT2-i); recent studies have in fact shown that the SGLT2-I dapaglifozin reduces per se albuminuria without increasing risk of hyperkalemia and AKI in diabetics with normal or mildly impaired renal function [78, 79]. Nevertheless, long-term trials are needed to verify the nephroprotective effectiveness of SGLT2-I in patients with overt CKD.

Therefore, it is possible to conclude that in proteinuric patients: (a) anti-RAAS therapy titrated to maximize reduction of proteinuria in CKD patients has a solid pathophysiological rationale and proven nephroprotective efficacy, and (b) in clinical practice, the treat-to-target approach is limited due to multiple-level escape mechanisms, as well as by side effects of therapy, the main being hyperkalemia.

### Downtitration of anti-RAAS as intervention in chronic HF or diabetic patients with CKD and hyperkalemia

RAAS blockade is the cornerstone of cardioprotection in chronic heart failure (CHF) and of renoprotection in diabetic kidney disease (DKD). Furthermore, RAAS blockers have shown to be effective in reversing cardiomyopathy and improving CV outcome of renal patients [80, 81]. However, there is concern about its systematic adoption in patients with reduced GFR because of the risk of worsening renal function and hyperkalemia [73].

NICE guidelines recommend not to offer anti-RAAS to CKD patients if pre-treatment sK is > 5.0 mEq/l, and to withdraw therapy when sK increases to 6.0 mEq/l [17]. In clinical practice, detection of sK > 5.5 mEq/l leads to a 5% dose reduction but even a 25% discontinuation of anti-RAAS in 194,456 outpatients included in the Geisinger Health System [8]. However, the problem is even more compelling for MRAs with a rate of discontinuation in up to half of patients administered this class of medication [8].

It is conceivable that downtitration or even withdrawal of anti-RAAS for safety reasons, although resulting in better control of sK, misses the opportunity of offering high risk patients the best available therapy for DKD or CHF. In fact, most CKD patients are not receiving adequate doses of these drugs due to hyperkalemia, and this is not to be underestimated, in that it is associated with increased incidence of adverse events and mortality [82]. This is especially true for MRAs that are used as add-on therapy for refractory CHF [83], and they have also shown to be effective in reducing proteinuria and slowing renal disease progression [84].

Indeed, NICE guidelines point out the importance of assessment, treatment, and eventual discontinuation of other factors or medications that can induce hyperkalemia in CKD high risk patients taking anti-RAAS [17]. Correction of metabolic acidosis by alkali assumption, and adherence to a low potassium diet could help in preventing hyperkalemia in these patients [85]. However, patient adherence is a major concern. Systematic use of diuretic therapy, especially in the outpatient setting management, could induce chronic hypovolemia and subsequent worsening of renal function, and chronic therapy with sodium polystyrene sulfonate (SPS) is burdened by side effects, often severe [86–88].

Therefore, even though downtitration or even withdrawal of anti-RAAS is a frequent strategy in the event of hyperkalemia in CKD patients with DKD and/or CHF, it is wise to offset other factors or to stop any other medication that can induce hyperkalemia.

### Pitfalls of current therapy of hyperkalemia in CKD

Careful monitoring of eGFR and sK levels should be regarded as the “core” of CKD management with anti-RAAS agents [82]. The two measures are indeed generally linked, due to the strict dependence of sK on GFR level. Treatment of hyperkalemia becomes even more important when considering that a mild-to-moderate reduction of GFR is expected when anti-RAAS therapy is started or uptitrated in proteinuric patients [30, 82, 89, 90].

**Fig. 2:**
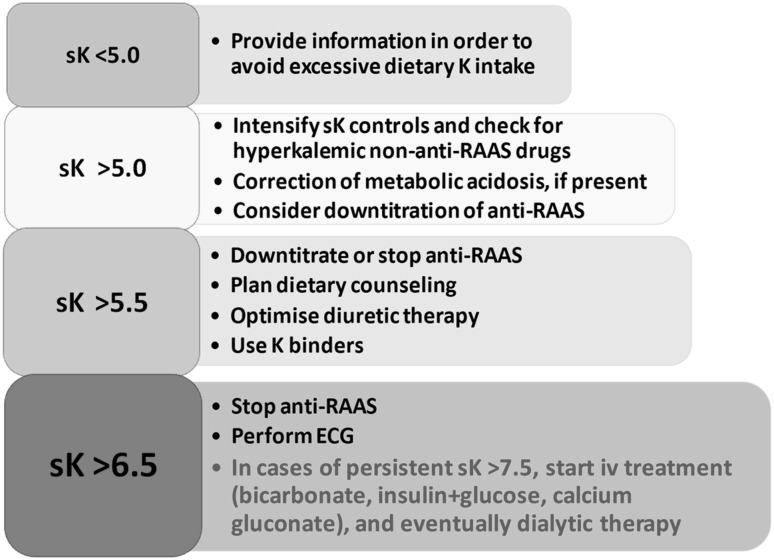
Stepwise approach to hyperkalemia in CKD patients under nephrology care. *K* potassium, *sK* serum potassium (mEq/l), *anti-RAAS* antagonists of renin–angiotensin–aldosterone system, *iv* intravenous, *ECG* electrocardiogram

A stepwise practical management approach to control sK levels in a renal clinic is depicted in Fig. 2. Treatment is multifaceted, with monitoring (including careful revision of drugs that potentially increase sK) and dietary counseling being the basic features. Oral supplementation of bicarbonate is only indicated in the presence of metabolic acidosis; similarly, more intensive control of glucose levels in diabetics is functional to favor cellular inflow of potassium with glucose. Patients will also take advantage from uptitration of the thiazide dose or a switch to loop diuretics correctly dosed according to eGFR value. Potassium-binding resins are generally prescribed when all the above interventions are not effective in controlling sK at levels ≤ 5.5 mEq/l. If levels persist above 6.5 mEq/l and symptoms (fatigue) and signs (ECG) develop, more aggressive therapy, including dialysis, is indicated.

Remarkably, however, the main interventions included in this “nephrologist” algorithm can often be ineffective.

#### Diet modification

Besides the common observation that patients are poorly compliant to prescribed changes in diet, available data do not support a consistent effect of restriction of potassium intake on sK levels [51]. On the other hand, a recent analysis of Modification of Diet in Renal Disease Study in non-diabetic patients with overt CKD has shown that higher dietary intake of potassium is associated with a lower risk for all-cause mortality [91]. Ad hoc clinical trials are therefore needed in CKD to verify whether restricting fruits/vegetables is truly effective in controlling sK and, more importantly, if it is associated with poor survival, as evidenced in the non-CKD population [92].

#### Diuretic therapy

Intensification of diuretic therapy in anti-RAAS treated patients is safe and efficacious only in the presence of discrete extracellular volume expansion; conversely, uptitrating diuretic therapy in hypovolemic or euvolemic patients can lead to excessive lowering of renal perfusion and function [93], which, in turn, causes an increase in sK levels. In this regard, strict monitoring of blood pressure can be helpful [94].

#### Potassium binding

SPS has been used in the last five decades as the cornerstone for the treatment of chronic hyperkalemia by inducing watery diarrhea with dependent potassium loss. However, the long term efficacy of SPS has not been evaluated in randomized, placebo-controlled trials. Moreover, it has been typically used in hospitals and less frequently in the outpatient setting due to low tolerability [86–88, 95]. In 2009, in fact, the US Food and Drug Administration posted a warning because of reported cases of colonic necrosis, bleeding, ischemic colitis, and perforation related to SPS [87]. Of note, in 2009 the FDA warned clinicians about adverse events occurring with SPS especially if premixed with sorbitol (that is the suspension commonly marketed in some countries) [96].

Calcium polystyrene sulfonate (CPS) is the other available K binder that has an advantage over SPS because, at variance with SPS, it avoids sodium retention by entrapping potassium in the distal colon in exchange for calcium. However, it is less used in nephrology practice than SPS, while it shares with SPS the paucity of data on usefulness and tolerability. Recently, a study was published on the long-term efficacy of oral CPS for treating mild hyperkalemia in 247 adult patients with baseline eGFR level of 30 ± 15 ml/min/1.73 m^2^ [97]. The findings suggested that CPS is effective and safe for controlling mild hyperkalemia in CKD. However, interpretation of this study is limited by the retrospective nature of the analysis, the use of electronic medical records, small sample size, inclusion of only Asian patients and the absence of any data on serum calcium.

The limitations of current treatment of hyperkalemia likely are the cause of the large number of anti-RAAS treated patients found to be hyperkalemic both in renal clinics and even in the ideal setting of randomized clinical trials. Therefore, there is an important unmet need for a well-tolerated therapy to control sK over the long term in CKD patients who require adequate RAAS blockade.

Therefore, the current approach to control sK in renal clinics is based on multi-target therapy. Aside from bicarbonate supplementation (indicated only in the presence of acidosis), the main components are the restriction of dietary potassium intake, intensive diuretic therapy and potassium binding. However, each of these three interventions is burdened by low efficacy and substantial side effects.

### Novel therapies to control sK in patients under anti-RAAS therapy

Until the FDA approval of patiromer in 2015 and the appearance of sodium zirconium cyclosilicate (ZS-9), the treatment of hyperkalemia in a chronic setting had remained unchanged for many years. These two new potassium-binding agents appear to have the potential to optimize control of chronic hyperkalemia and therefore enable optimal anti-RAAS therapy (Table 3). Here we report the main results of trials on the two new drugs, that can be useful for the clinical nephrologist while awaiting real-world data on a larger number of patients treated for a longer period of time.

**Table 3:** Characteristics of potassium-binding agents for the treatment of hyperkalemia

Characteristic	SPS [86–88]	Patiromer [98, 101, 102]	ZS-9 [103, 104]
Molecule	Nonspecific, sodium-containing, organic resin	Selective, sodium-free, organic polymer	Selective, sodium-containing, inorganic crystalline silicate
Mechanism of action	Nonspecific binding of potassium in exchange for sodium	Nonspecific binding of potassium in exchange for calcium	Selective potassium binding in exchange for sodium
Administration/formulation	Oral or rectal suspension	Oral suspension	Oral suspension
Site of action	Colon	Distal colon	Entire intestinal tract
Onset of effect	1–2 h	7 h	1 h
Dosing	15–60 g/day orally; 30–50 g/day rectally	8.4–25.2 g/day	2.5–30 g/day depending on the acute/chronic situation
Common adverse events	Gastrointestinal disorders Hypernatremia Hypokalemia Alkalosis Volume overload	Gastrointestinal disorders Hypokalemia Hypomagnesemia	Gastrointestinal disorders Hypokalemia Edema
Serious adverse events	Colonic necrosis	None	None

*GI* gastrointestinal, *SPS* sodium polystyrene sulfonate, *ZS-9* sodium zirconium cyclosilicate

### Patiromer

Patiromer is a non-absorbed polymer that binds potassium in exchange for calcium throughout the gastrointestinal tract, resulting in an increase in intestinal secretion of potassium with a consequent reduction of sK levels. Patiromer is stable and nonsystemically absorbed and has been extensively studied in patients with CKD and/or cardiovascular disease [98].

The phase 3 study conducted by Weir et al. was designed to examine CKD patients with hyperkalemia (sK 5.1–6.5 mEq/l) treated with anti-RAAS, and randomized to patiromer or placebo [21]. A significant reduction in sK in the patiromer arm was observed in the first 4 weeks. The second phase of the study involved patients with baseline sK 5.5–6.5 mEq/l; levels decreased to 3.8–5.1 mEq/l following treatment with patiromer. These patients then entered into an 8-week randomized withdrawal phase in which they either continued on patiromer or switched to placebo. In this phase, a significant difference in sK change (0.72 mEq/l increase in placebo vs. 0 in patiromer) was observed. It is worth noting that over 90% of patients with sK > 5.5 mEq/l at baseline were able to remain on anti-RAAS therapy, while less than 50% of patients who were switched to placebo maintained the RAAS-blocking drug [21].

Interestingly, more than half the study participants had diabetes, eGFR < 30 ml/min/1.73 m^2^ and 40% had CHF. The mean age of patients enrolled in the study was nearly 65 years. Therefore, the study population was a high-risk group that was successfully maintained on the indicated anti-RAAS therapy. Patiromer was well tolerated throughout the study, constipation being the only adverse event with a reported frequency of > 3%.

Similar effects have been demonstrated in a long-term clinical trial in patients with type 2 diabetes [22]. Interestingly, a significant reduction in mean systolic and diastolic blood pressure was also observed in this trial. This effect could be related to the maintenance of RAAS-blocking therapy, better adherence to antihypertensive therapy during the course of the study or the possibility that patiromer may be associated with intestinal sodium binding. The blood pressure-lowering effect is supported by the phase 3 OPAL-HK study where patients randomized to patiromer had a significant reduction in mean systolic/diastolic blood pressure in association with decreasing aldosterone levels [99]. Nonetheless, in CKD patients, higher K excretion (testifying higher intake) is associated with increased risk of CKD progression [100]. Therefore, future studies on the novel K-binders indicated for chronic therapy should verify the long-term effects on CKD progression. Hypomagnesemia was the most common treatment-related adverse event (7.2%), but no patient developed severe hypomagnesemia.

Patiromer has also been studied to determine its onset of action in patients with CKD receiving anti-RAAS treatment [101]. After a 3-day potassium- and sodium-restricted diet in an inpatient general clinical research unit, 25 patients with sustained hyperkalemia (5.5–6.4 mEq/l) received patiromer 8.4 g twice daily. Mean baseline sK was 5.93 mEq/l, and decreased significantly 7 h after the first dose of the drug and at all subsequent assessments through 48 h. At 48 h (14 h after the last dose), the mean reduction was − 0.75 mEq/l [101]. Of note, due to potential drug–drug interactions, the administration of other oral medications should be at least 3 h before or 3 h after administration of patiromer [102].

### ZS-9

ZS-9 is an insoluble, highly stable and non-systemically absorbed inorganic sodium–potassium cation exchange drug, currently undergoing clinical evaluation as a potential treatment for hyperkalemia [103, 104]. Two phase 3 trials have been completed, and a third is currently investigating long-term therapy. Overall, these studies have demonstrated that ZS-9 is an effective potassium binder that corrects hyperkalemia with a dose-dependent effect within 48 h of treatment. The reduction in sK is most pronounced in patients with higher baseline levels, and ZS-9 is effective in normalizing kalemia in patients, whether or not they are receiving anti-RAAS therapy [20, 103]. Adverse events have been found to be comparable in ZS-9 and placebo, although edema is more frequent in patients treated with the drug.

Patiromer and ZS-9 therefore appear to be effective and well tolerated drugs that can be used to reduce sK levels consistently and safely. The recent FDA approval of patiromer, and the expected approval of ZS-9, should allow an opportunity for clinicians to use recommended and appropriate doses of anti-RAAS agents in patients with CKD and cardiovascular disease, particularly allowing a wider use of MRAs that may have important additive benefits when administered in association with traditional RAAS-blocking drugs.

It is worth noting that European guidelines for the diagnosis and treatment of HF have recently highlighted the results of patiromer and ZS-9 studies in patients with CHF as they confirm the efficacy of these new therapies in reducing sK and preventing recurrent hyperkalemia in patients with CHF and CKD, in the context of anti-RAAS treatment [83].
